# DIMP53‐1: a novel small‐molecule dual inhibitor of p53–MDM2/X interactions with multifunctional p53‐dependent anticancer properties

**DOI:** 10.1002/1878-0261.12051

**Published:** 2017-05-02

**Authors:** Joana Soares, Margarida Espadinha, Liliana Raimundo, Helena Ramos, Ana Sara Gomes, Sara Gomes, Joana B. Loureiro, Alberto Inga, Flávio Reis, Célia Gomes, Maria M. M. Santos, Lucília Saraiva

**Affiliations:** ^1^ UCIBIO/REQUIMTE Universidade do Porto Portugal; ^2^ Laboratório de Microbiologia Departamento de Ciências Biológicas Faculdade de Farmácia Universidade do Porto Portugal; ^3^ Research Institute for Medicines (iMed.ULisboa) Faculty of Pharmacy Universidade de Lisboa Portugal; ^4^ CIBIO Centre for Integrative Biology Laboratory of Transcriptional Networks University of Trento Italy; ^5^ Laboratory of Pharmacology & Experimental Therapeutics Institute for Biomedical Imaging and Life Sciences (IBILI) Faculty of Medicine University of Coimbra Portugal; ^6^ Centre for Neuroscience and Cell Biology – Institute for Biomedical Imaging and Life Sciences (CNC.IBILI) Research Consortium University of Coimbra Portugal

**Keywords:** anticancer therapy, MDM2, MDMX, p53, small‐molecule, tryptophanol‐derived oxazoloisoindolinone

## Abstract

The transcription factor p53 plays a crucial role in cancer development and dissemination, and thus, p53‐targeted therapies are among the most encouraging anticancer strategies. In human cancers with wild‐type (wt) p53, its inactivation by interaction with murine double minute (MDM)2 and MDMX is a common event. Simultaneous inhibition of the p53 interaction with both MDMs is crucial to restore the tumor suppressor activity of p53. Here, we describe the synthesis of the new tryptophanol‐derived oxazoloisoindolinone DIMP53‐1 and identify its activity as a dual inhibitor of the p53–MDM2/X interactions using a yeast‐based assay. DIMP53‐1 caused growth inhibition, mediated by p53 stabilization and upregulation of p53 transcriptional targets involved in cell cycle arrest and apoptosis, in wt p53‐expressing tumor cells, including MDM2‐ or MDMX‐overexpressing cells. Importantly, DIMP53‐1 inhibits the p53–MDM2/X interactions by potentially binding to p53, in human colon adenocarcinoma HCT116 cells. DIMP53‐1 also inhibited the migration and invasion of HCT116 cells, and the migration and tube formation of HMVEC‐D endothelial cells. Notably, in human tumor xenograft mice models, DIMP53‐1 showed a p53‐dependent antitumor activity through induction of apoptosis and inhibition of proliferation and angiogenesis. Finally, no genotoxicity or undesirable toxic effects were observed with DIMP53‐1. In conclusion, DIMP53‐1 is a novel p53 activator, which potentially binds to p53 inhibiting its interaction with MDM2 and MDMX. Although target‐directed, DIMP53‐1 has a multifunctional activity, targeting major hallmarks of cancer through its antiproliferative, proapoptotic, antiangiogenic, anti‐invasive, and antimigratory properties. DIMP53‐1 is a promising anticancer drug candidate and an encouraging starting point to develop improved derivatives for clinical application.

**Abbreviations**
CETSA: cellular thermal shift assay
Co‐IP: co‐immunoprecipitation
DMSO: dimethyl sulfoxide
MDM: murine double minute
MVD: microvessel densities
SRB: sulforhodamine B
VEGF: vascular endothelial growth factor
wt: wild‐type

## 1 Introduction

The sequence‐specific transcription factor p53 regulates a plethora of genes involved in crucial cellular processes, including cell cycle arrest, cell death, and DNA repair (Hong *et al*., 2014). Inactivation of the p53 tumor suppressor function is a common event in human cancers with a dramatic impact on tumor development and dissemination (Burgess *et al*., 2016; Hong *et al*., 2014; Wade *et al*., 2013). Although a substantial proportion of cancers harbor wild‐type (wt) p53, its function is found inactivated or at least inhibited (Burgess *et al*., 2016; Wade *et al*., 2013), mainly by the murine double minute (MDM) proteins, MDM2 and MDMX (or MDM4). Mechanistically, both MDMs bind to p53 inhibiting its transcriptional activity. Additionally, the E3 ligase MDM2 triggers p53 ubiquitin‐proteasome degradation. Although MDMX has no E3 ligase activity, the MDM2–MDMX heterodimer ubiquitinates p53 with higher efficiency than MDM2 homodimers (Burgess *et al*., 2016; Gomes *et al*., 2016; Wade *et al*., 2013). Therefore, both MDMs are powerful oncogenes, commonly overexpressed in several human cancers (Burgess *et al*., 2016).

Accumulating data demonstrate that wt p53 is a valuable therapeutic target and that its activation through inhibition of the p53–MDM interactions is a promising anticancer strategy. Many p53–MDM2 interaction inhibitors have been identified, several of which are currently under clinical trials (Burgess *et al*., 2016; Gomes *et al*., 2016; Wade *et al*., 2013). Nonetheless, given the distinct and cooperative function of both MDMs on p53 inactivation (Burgess *et al*., 2016; Gomes *et al*., 2016; Wade *et al*., 2013), and the resistance of MDMX‐overexpressing cells to MDM2‐only inhibitors (e.g., Nutlin‐3a) (Li and Lozano, 2013), small molecules that suppress the inhibitory effect of both MDMs represent the ideal strategy for full p53 reactivation (Burgess *et al*., 2016; Gomes *et al*., 2016; Wade *et al*., 2013). However, the availability of such compounds is still limited (Graves *et al*., 2012; Lee *et al*., 2011; Soares *et al*., 2015a).

Here, we report the identification of DIMP53‐1 as a new p53 activator, which potentially binds to p53 inhibiting its interaction with MDM2 and MDMX. Additionally, DIMP53‐1 has *in vitro* and *in vivo* p53‐dependent antitumor properties, involving antiproliferative, proapoptotic, antiangiogenic, anti‐invasive, and antimigratory activities.

## 2 Materials and methods

### 2.1 Reagents

Nutlin‐3a, SJ‐172550, etoposide, cycloheximide, and cyclophosphamide were from Sigma‐Aldrich (Sintra, Portugal). All tested compounds were dissolved in dimethyl sulfoxide (DMSO; Sigma‐Aldrich). Primary antibodies used in western blot and immunohistochemistry were from Santa Cruz Biotechnology (Frilabo, Porto, Portugal; mouse monoclonal anti‐p53, anti‐MDM2, anti‐BAX, anti‐PARP, anti‐PUMA, anti‐GAPDH, and rabbit polyclonal anti‐p21), Bethyl Laboratories (Montgomery, TX, USA; rabbit polyclonal anti‐MDMX), Invitrogen (Alfagene, Carcavelos, Portugal; mouse monoclonal anti‐Pgk1p), Abcam (Cambridge, UK; rabbit monoclonal antihistone H2AX, phospho S139), and Pierce Thermo Scientific (Taper, Sintra, Portugal; mouse monoclonal anti‐VEGF, anti‐CD34, and rabbit monoclonal anti‐Ki‐67). Secondary antibodies anti‐mouse and anti‐rabbit horseradish peroxidase‐conjugated were from Santa Cruz Biotechnology (Frilabo).

### 2.2 Chemical synthesis of DIMP53‐1

**Figure 1:**
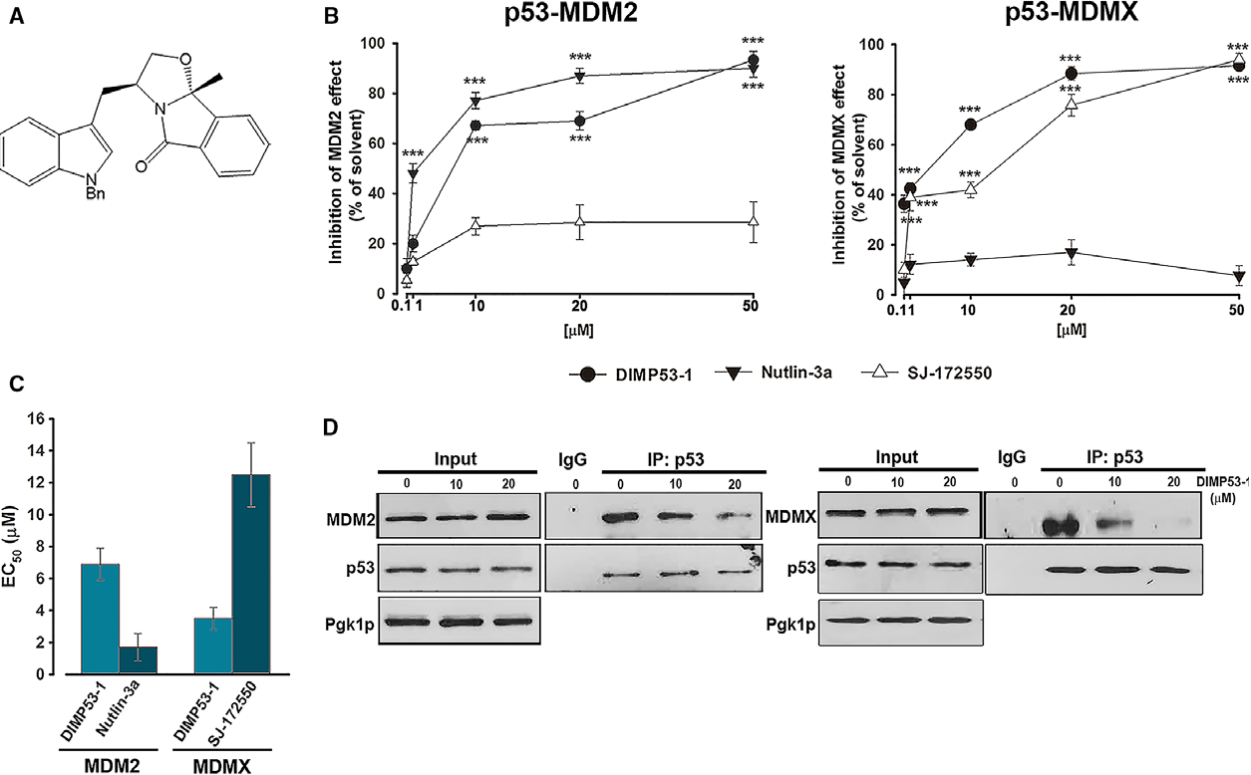
Identification of DIMP53‐1 as a potential dual inhibitor of the p53–MDM2/X interactions using yeast. (A) DIMP53‐1 chemical structure. (B) Effect of 0.1–50 μm DIMP53‐1, Nutlin‐3a, and SJ‐172550 on the reestablishment of p53‐induced growth inhibition in MDM2/MDMX‐co‐expressing yeast, for 42 h; results were plotted setting the growth of DMSO‐treated cells expressing p53 alone as 100%; data are mean ± SEM of six independent experiments; values were significantly different from DMSO (***P <* *0.001). (C) EC_50_ values of DIMP53‐1, Nutlin‐3a, and SJ‐172550 obtained from concentration–response curves presented in (B). (D) Co‐IP was performed with anti‐p53 (IP:p53) or anti‐immunoglobulin G (IgG) antibodies, followed by immunoblotting with anti‐MDM2, anti‐MDMX, and anti‐p53 antibodies in cells treated with 10 and 20 μm of DIMP53‐1 or DMSO for 42 h; whole‐cell lysate (input).

Synthesis of DIMP53‐1 (Fig. 1A) (Pereira *et al*., 2015; Soares *et al*., 2016) is described in Supporting information (Scheme S1; Fig. S1).

### 2.3 Yeast‐based screening assay

*Saccharomyces cerevisiae* cells expressing human wt p53 alone or co‐expressed with human MDM2/MDMX were used, as described (Soares *et al*., 2015a). Briefly, cells were grown in galactose‐selective medium with compounds or 0.1% DMSO for 42 h; cell growth was analyzed by colony‐forming unit counts with the determination of EC_50_ (concentration that causes 50% of effect) values.

### 2.4 Human cell lines and growth conditions

Colon adenocarcinoma HCT116 cell lines expressing wt p53 (HCT116p53^+/+^) and its p53‐null isogenic derivative (HCT116p53^−/−^) were provided by B. Vogelstein (The Johns Hopkins Kimmel Cancer Center, Baltimore, MD, USA): breast adenocarcinoma MCF‐7, osteosarcoma SJSA‐1, and nontumorigenic breast epithelial MCF10A cell lines (ATCC, LGC Standards S.L.U., Barcelona, Spain, 2013); dermal microvascular endothelial HMVEC‐D cells (Lonza, VWR, Carnaxide, Portugal, 2013). Tumor cells were cultured in RPMI‐1640 with UltraGlutamine (Lonza, VWR), supplemented with 10% fetal bovine serum (FBS; Gibco, Alfagene). MCF10A cells were cultured in DMEM/F‐12 supplemented with MEGM SingleQuot Kit Suppl. & Growth Factors (Lonza, VWR). HMVEC‐D cells were cultured in endothelial cell growth basal medium supplemented with SingleQuots™ Kit (Lonza, VWR). Cell lines were maintained in a humidified incubator at 37 °C with 5% CO_2_.

### 2.5 Co‐immunoprecipitation (Co‐IP) assay

Co‐IP was performed using the Pierce Classic Magnetic IP and Co‐IP Kit (Thermo Scientific, Dagma, Carcavelos, Portugal), as described (Soares *et al*., 2015a). Detection of p53, MDM2, MDMX, and GAPDH was performed by western blot.

### 2.6 Sulforhodamine B (SRB) assay

Cell lines were seeded in 96‐well plates at the cells/well density of 5.0 × 10^3^ (for HCT116, MCF‐7, and SJSA‐1) and 1.0 × 10^4^ (for MCF10A and HMVEC‐D). Cells were thereafter treated with serial dilutions (1.85–75 μm) of DIMP53‐1 for 24 and 48 h, and its effect on cell proliferation was analyzed by sulforhodamine B (SRB) assay (Soares *et al*., 2015a) with the determination of IC_50_ (concentration that causes 50% of growth inhibition) values. The solvent (DMSO 0.25%) was included as control.

### 2.7 Cell cycle and apoptosis

HCT116, MCF‐7, and SJSA‐1 cells were seeded in six‐well plates at 1.5 × 10^5^ cells/well density. Cells were thereafter treated with DIMP53‐1 or solvent for 24 h. For cell cycle, cells were stained with propidium iodide (PI; Sigma‐Aldrich) followed by flow cytometric analysis (Soares *et al*., 2015a). For apoptosis, cells were analyzed by flow cytometry using the Annexin V‐FITC Apoptosis Detection Kit I (BD Biosciences, Enzifarma, Porto, Portugal) according to the manufacturer's instructions (Soares *et al*., 2015a).

### 2.8 RNA extraction and RT‐qPCR

Total RNA, from HCT116 cells treated with DIMP53‐1 or solvent for 24 h, was extracted using the Illustra™ RNAspin Mini RNA Isolation Kit (GE Healthcare Life Sciences, Milan, Italy). One microgram of RNA was used for cDNA synthesis using the M‐MuLV reverse transcriptase and RevertAid cDNA Synthesis kit (ThermoFisher, Monza and Brianza, Italy) in 20 μL final volume, following the manufacturer's instructions. RT‐qPCR assays were performed in a 384‐well plate on a CFX Touch Real‐Time PCR Detection System (Bio‐Rad, Milan, Italy), starting with 25 ng of cDNA (Lion *et al*., 2013). The 2X KAPA SYBR^®^ FAST qPCR Kit (Kapa Biosystems, Rome, Italy) and specific primers for *BAX* and *CDKN1A* (p21), Eurofins (MWG, Milan, Italy), were used; *GAPDH* and *B2M* were used as reference genes.

### 2.9 Western blot

HCT116, MCF‐7, and SJSA‐1 cells were seeded in six‐well plates at 1.5 × 10^5^ cells/well density. After treatment with compounds or solvent, cells were lysed and the protein fractions were analyzed by western blot, as described (Soares *et al*., 2015b). Antibodies are described in 2.1.

### 2.10 Cellular Thermal Shift Assay (CETSA)

To evaluate drug target interactions in cells, the cellular thermal shift assay (CETSA) was performed as described (Tan *et al*., 2015). Briefly, HCT116p53^+/+^ cells were lysed in appropriate buffer (25 mm Tris pH 7.4, 10 mm MgCl_2_, 2 mm DTT) by Dounce homogenization. HCT116p53^+/+^ cell lysates were incubated with DIMP53‐1 or solvent for 1 h at room temperature and then heated to the indicated temperatures for 3 min, cooled to room temperature for 3 min, and placed on ice. Insoluble protein was separated by centrifugation, and soluble protein was detected by western blot. In the experiments at different heating temperatures, the signal intensity was normalized to the intensity at 25 °C (GAPDH denaturation with heating temperatures unable to use as loading control). At a constant temperature (39 °C), the increase in nondenatured p53 was calculated setting the signal obtained with DMSO at 39 °C as 0, and the signal obtained with DMSO at 25 °C (considered the maximum amount of nondenatured p53) as 1.

### 2.11 *In vitro* migration and invasion assays

Cell migration was analyzed using the wound healing assay and the QCM 24‐Well Fluorimetric Chemotaxis Cell Migration Kit (8 μm; Merck Millipore, VWR), as described (Soares *et al*., 2016). In the wound healing assay, confluent HCT116p53^+/+^ and HMVEC‐D cells, with a wound in the middle of the well, were treated with 3 μm DIMP53‐1 or solvent. Cells were photographed using the Moticam 5.0MP camera with Motic's AE2000 inverted microscope with 400× magnification at different time‐points of treatment until complete closure of the wound. Wound closure was calculated by subtracting the ‘wound’ area (measured using image j software, Bethesda, MD, USA) at the indicated time periods after treatment from the initial (0 h) ‘wound’ area. In Chemotaxis Cell Migration Kit, 0.5 × 10^6^ cells·mL^−1^ of HCT116p53^+/+^ cells were prepared in serum‐free RPMI 1640 and treated with 3 μm of DIMP53‐1 or solvent, for 24 h. The prepared cell suspensions were distributed in 24‐well plates (300 μL/insert), followed by the addition of 500 μL medium containing 10% FBS to the lower chamber. Cells that migrated through the 8‐μm pore membranes were eluted, lysed, and stained with a green fluorescence dye that binds to cellular nucleic acids. Cell invasion was analyzed using the QCM 24‐Well Fluorimetric Cell Invasion Kit (Merck Millipore, VWR), according to the manufacturer's instructions. This assay consists in the evaluation of the capacity of cell migration through the ECMatrix layer of the upper chamber of the system, and it was performed as chemotaxis cell migration assay, except the incubation time with DIMP53‐1 or solvent of 48 h. In both assays, the number of migrated cells was proportional to the fluorescence signal measured using the Bio‐Tek Synergy HT plate reader (Izasa, Gondomar, Portugal) at 480/520 nm (excitation/emission).

### 2.12 Angiogenesis assay

Endothelial tube formation was evaluated using the *in vitro* Angiogenesis Assay Kit (Millipore, VWR) according to the manufacturer's instructions. Briefly, 3 × 10^4^ HMVEC‐D cells/well were seeded in 24‐well plates coated with ECMatrix with DIMP53‐1 or solvent for 16 h. Cells were photographed (Moticam 5.0MP camera; Motic's AE2000 microscope, VWR).

### 2.13 Comet assay

DNA damage was evaluated in HCT116p53^+/+^ cells, after 48‐h treatment with 7, 14, and 21 μm DIMP53‐1, 25 μm etoposide (positive control), or solvent, using the OxiSelect Comet Assay kit (Cell Biolabs, MEDITECNO, Carcavelos, Portugal), according to the manufacturer's instructions, with TBE (Tris/borate/EDTA) for electrophoresis. Cells were photographed (Nikon DS‐5Mc camera; Nikon Eclipse E400 fluorescence microscope; Nikon act‐2u software, Izasa).

### 2.14 Micronucleus assay

Genotoxicity was analyzed by the cytokinesis‐block micronucleus assay in human lymphocytes, as described (Soares *et al*., 2016). Briefly, fresh peripheral blood samples were collected from healthy volunteers into heparinized vacutainers. Blood samples, suspended in RPMI medium supplemented with 10% FBS, were treated with 7, 14, and 21 μm of DIMP53‐1, 1 μg·mL^−1^ cyclophosphamide (positive control), or solvent for 44 h. Cells were thereafter treated with 3 μg·mL^−1^ cytochalasin B (cytokinesis preventive) for 28 h. Lymphocytes were isolated by density gradient separation (Histopaque‐1077 and ‐1119), fixed in 3 : 1 methanol/glacial acetic acid, and stained with Wright stain. For each sample, 1000 binucleated lymphocytes were blindly scored using a Leica light optical microscope (Wetzlar, Germany); the number of micronuclei per 1000 binucleated lymphocytes was recorded.

### 2.15 *In vivo* antitumor and toxicity assays

Animal experiments were conducted according to the EU Directive 2010/63/EU and to the National Authorities. The BALB/c nude mice and Wistar rats (Charles‐River Laboratories, Barcelona, Spain) were housed under pathogen‐free conditions in individual ventilated cages. For toxicity assays, Wistar rats were treated with 50 mg·kg^−1^ DIMP53‐1, vehicle (DMSO), or saline solution (control) by intraperitoneal injection, twice a week, for two weeks. After four administrations, blood samples and organs (kidneys, spleen, heart, and liver) were collected for toxicological analysis. Each group was composed of four animals. Xenograft tumor assays were performed with HCT116p53^+/+^ and HCT116p53^−/−^ tumor cells. Briefly, 1 × 10^6^ HCT116 cells (in PBS) were inoculated subcutaneously in the mice dorsal flank. Tumor dimensions were assessed by caliper measurement, and their volumes were calculated [tumor volume = (*L *× *W* ^2^)/2], where *L* and *W* represent the longest and shortest axis of the tumor, respectively. Treatment started when tumors reached approximately 100 mm^3^ volume (14 days after the grafts). Mice were thereafter treated twice a week with 50 mg·kg^−1^ DIMP53‐1 or vehicle by intraperitoneal injection for two weeks. Tumor volumes and body weights were monitored twice a week until the end of the treatment. Animals were sacrificed by cervical dislocation at the end of the study, when tumors reached 1500 mm^3^ or if the animals presented any signs of morbidity. Each group was composed of six animals.

### 2.16 Immunohistochemistry

Tumor tissues were fixed in 10% formalin, embedded in paraffin, sectioned at 4 μm, and stained with hematoxylin and eosin (H&E) or antibodies, as described (Soares *et al*., 2016). Briefly, antigen retrieval was performed by boiling the sections for 20 min in 10 mm citrate buffer (pH 6.0) for staining with all antibodies, except for anti‐VEGF for which tissues were treated with 10 mm EDTA buffer (pH 8.0). Antibodies are described in 2.1. Immunostaining was carried out using the UltraVision Quanto Detection System HRP DAB Kit, from Lab Vision Thermo Scientific, according to the manufacturer's instructions. Evaluation of DAB (3,3′‐diaminobenzidine) intensity and quantification of marked cells were performed using image j software. Microvessel densities were determined by counting CD34‐positive vessels, as described (Maeda *et al*., 2007; Weidner *et al*., 1991).

### 2.17 TUNEL assay

TUNEL assay was performed using the *In Situ* Cell Death Detection Kit Fluorescein (Roche, Sigma‐Aldrich), according to the manufacturer's instructions, as described (Soares *et al*., 2016).

### 2.18 Flow cytometric data acquisition and analysis

The Accuri™ C6 flow cytometer and the cellquest software (BD Biosciences, Enzifarma) were used. The flowjo software (Ashland, OR, USA) was used to identify and quantify cell cycle phases.

### 2.19 Statistical analysis

Data were statistically analyzed using the graphpad prism software (La Jolla, CA, USA). Differences between means were tested for significance using Student's *t*‐test (*P <* *0.05; **P <* *0.01; ***P <* *0.001).

## 3 Results

### 3.1 Identification of DIMP53‐1 as a potential dual inhibitor of the p53–MDM2/X interactions using a yeast‐based assay

Derivatives of SLMP53‐1, a tryptophanol‐derived oxazoloisoindolinone p53 activator (Soares *et al*., 2016) [International Patent (Soares *et al*., 2014)], containing different protective groups in the nitrogen of the indole moiety, were synthesized. The effect of this new chemical library on p53–MDM2 and p53–MDMX interactions was thereafter investigated, using the reported yeast‐based screening assay (Soares *et al*., 2015a). In this assay, the expression of human wt p53 in yeast causes growth arrest that is inhibited by human MDM2 or MDMX. The effect of 0.1–50 μm compounds was evaluated, and DIMP53‐1 (Fig. 1A) was identified as a potential dual inhibitor of the p53–MDM2/X interactions (Fig. 1B). Nutlin‐3a and SJ‐172550 were used as positive controls because, as in human cells (Reed *et al*., 2010; Vassilev *et al*., 2004), they inhibit the negative effect of MDM2 and MDMX, respectively, having no impact on the other MDM (Fig. 1B). Contrary to Nutlin‐3a and SJ‐172550, DIMP53‐1 relieved the negative effect of both MDMs on p53 (Fig. 1B). Based on EC_50_ values, DIMP53‐1 was less effective than Nutlin‐3a on p53–MDM2 interaction, but more effective than SJ‐172550 on p53–MDMX interaction (Fig. 1C). Additionally, 0.1–50 μm DIMP53‐1 did not interfere with the growth of control yeast (data not shown), corroborating its selectivity toward the p53–MDM2/X interactions.

The ability of DIMP53‐1 to block the p53–MDM2/X interactions in yeast was further demonstrated by Co‐IP (Fig. 1D). Actually, 10 and 20 μm DIMP53‐1 led to a visible decrease in the amount of MDM2 or MDMX co‐immunoprecipitated with p53 in yeast cells co‐expressing p53 and MDM2 or MDMX, respectively.

### 3.2 DIMP53‐1 causes p53 stabilization and upregulation of p53 transcriptional targets through potential binding to p53, inhibiting its interaction with MDM2 and MDMX, in human tumor cells

**Figure 2:**
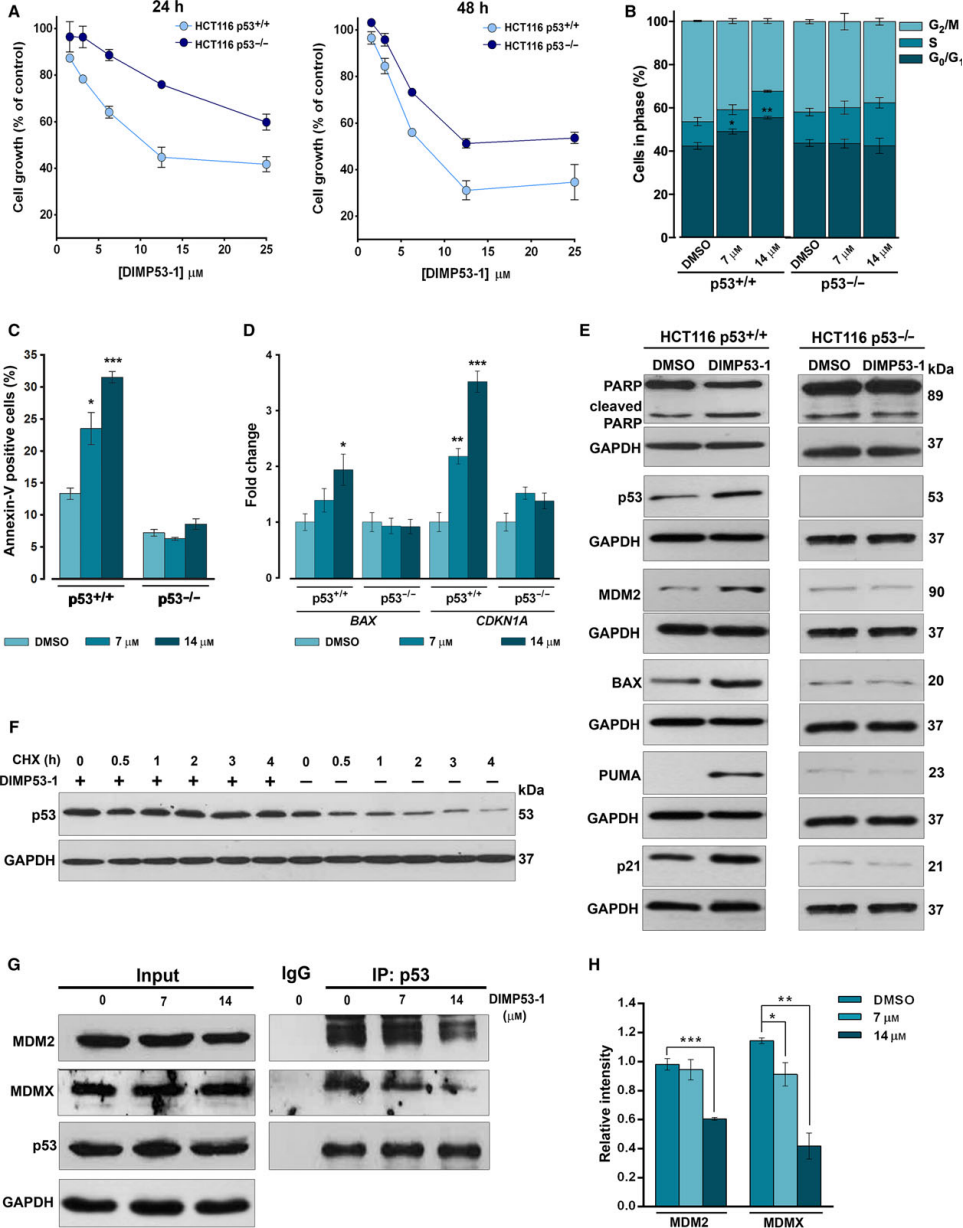
DIMP53‐1 shows a p53‐dependent growth inhibitory effect in human tumor cells mediated by cell cycle arrest, apoptosis, p53 stabilization, upregulation of p53 target genes, and disruption of the p53–MDM2/X interactions. (A) Concentration–response growth curves for DIMP53‐1 in p53^+/+^ and p53^−/−^ HCT116, after 24‐ and 48‐h treatments; data are mean ± SEM of four independent experiments; incubation with DMSO, in equivalent % of DIMP53‐1, was used to normalize the results. (B,C) Cell cycle arrest (B) and apoptosis (C) were determined at 7 and 14 μm of DIMP53‐1 for 24 h in p53^+/+^ and p53^−/−^ HCT116 cells; data are mean ± SEM of three independent experiments; values were significantly different from DMSO (*P <* *0.05; **P <* *0.01; ***P <* *0.001). (D) mRNA levels of *BAX* and *CDKN1A* (p21) after 24‐h treatment with 7 and 14 μm of DIMP53‐1 in p53^+/+^ and p53^−/−^ HCT116 cells; fold expression changes are relative to DMSO and correspond to mean ± SEM of three independent experiments. (E) Western blot analysis was performed after 24‐h (MDM2, p53) and 48‐h (PARP, BAX, PUMA, p21) treatments with 7 μm of DIMP53‐1 or DMSO in p53^+/+^ and p53^−/−^ HCT116 cells. (F) p53 protein levels in HCT116p53^+/+^ cells treated for 24 h with DIMP53‐1 or solvent followed with cycloheximide (CHX; 150 μg/mL). (G) Co‐IP was performed with anti‐p53 (IP:p53) or anti‐immunoglobulin G (IgG) antibodies, followed by immunoblotting with anti‐MDM2, anti‐MDMX, and anti‐p53 antibodies in HCT116p53^+/+^ cells treated with 7 and 14 μm of DIMP53‐1 or DMSO for 8 h; whole‐cell lysate (input); in IP:p53 of MDM2, the cut top band corresponds to the anti‐p53 antibody, and the other two bands correspond to MDM2 isoforms. (H) Quantification of IP:p53 immunoblots; data are mean ± SEM of three independent experiments; values were significantly different from DMSO (*P <* *0.05; **P <* *0.01; ***P <* *0.001). In (E), (F), and (G), immunoblots are representative of three independent experiments; GAPDH was used as loading control.

To confirm the molecular mechanism of action of DIMP53‐1 as a dual inhibitor of the p53–MDM2/X interactions, its activity was ascertained in p53^+/+^ and p53^−/−^ HCT116 cells. The SRB assay revealed a significant reduction in the DIMP53‐1 growth inhibitory effect in the absence of p53, during 24‐ and 48‐h treatment (Fig. 2A). Despite the selectivity of DIMP53‐1 to the p53‐pathway, this compound also inhibited the growth of HCT116p53^−/−^ cells. This may indicate an alternative mechanism of action of DIMP53‐1 independent of p53, for longer incubation times and higher concentrations of compound.

In HCT116p53^+/+^ cells, 7 and 14 μm of DIMP53‐1‐induced growth inhibition were associated with G0/G1‐phase cell cycle arrest (Fig. 2B) and apoptosis, as evidenced by the increase in Annexin V‐positive cells (Fig. 2C) and PARP cleavage (Fig. 2E), not observed in p53‐null HCT116 cells. Accordingly, 7 μm of DIMP53‐1 upregulated major p53 transcriptional targets, as revealed by the increased *BAX* and *CDKN1A* (p21) mRNA levels (Fig. 2D), and MDM2, BAX, PUMA, and p21 protein levels (Fig. 2E) in p53^+/+^, but not in p53^−/−^, HCT116 cells. Interestingly, in HCT116p53^+/+^ cells, the slight increase in p21 expression levels is in accordance with the modest cell cycle arrest induced by DIMP53‐1. Additionally, by cycloheximide treatment to block the protein synthesis, it was confirmed that DIMP53‐1 increased the half‐life of p53, due to p53 stabilization (Fig. 2F).

**Figure 3:**
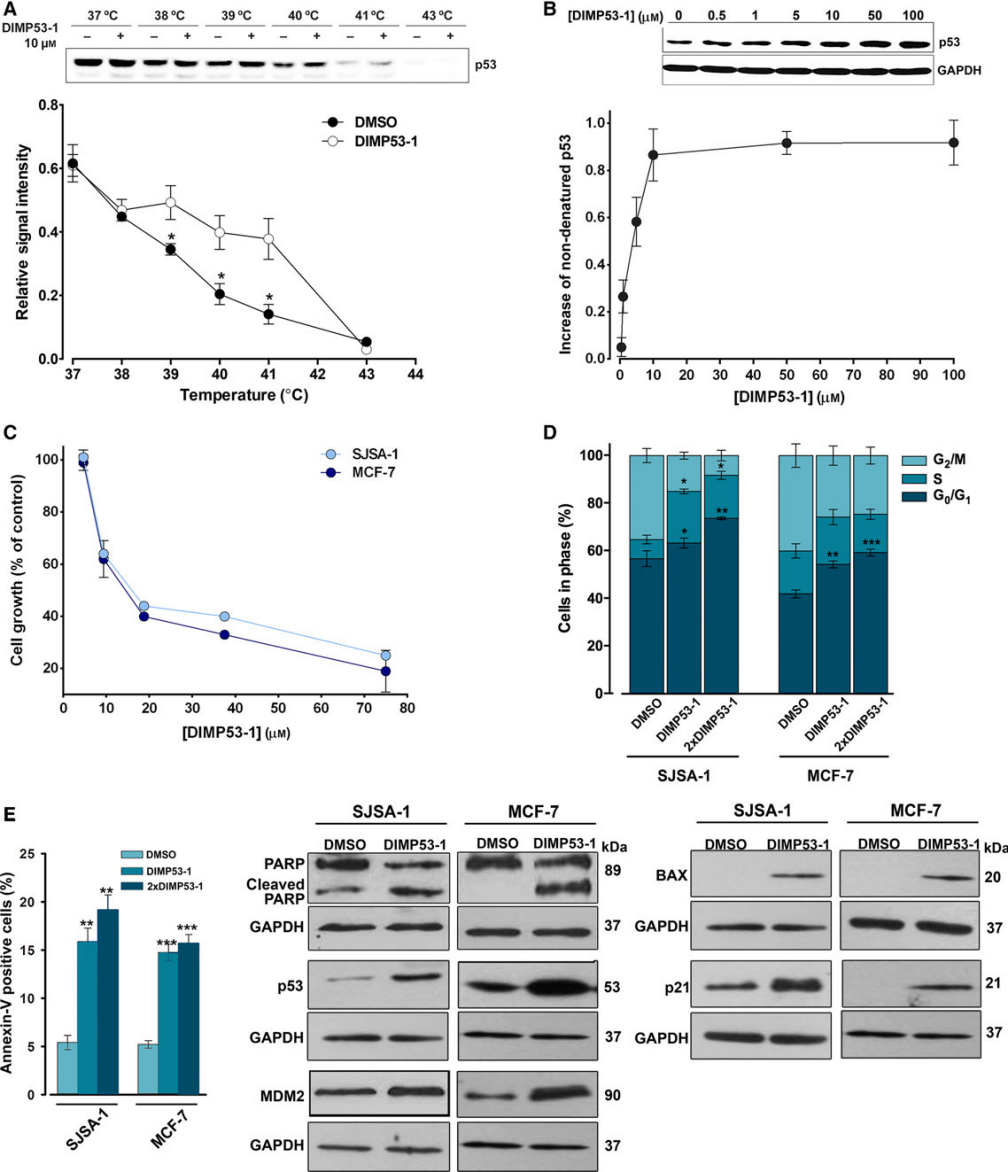
DIMP53‐1 potentially binds to p53 and inhibits the growth of MDM2‐ and MDMX‐overexpressing tumor cells through induction of cell cycle arrest, apoptosis, and upregulation of p53 target genes. (A and B) CETSA experiments were performed in HCT116p53^+/+^ cell lysates in the presence or absence of DIMP53‐1. In (A), 10 μm of DIMP53‐1 was used and lysate samples were heated at different temperatures; plot represents the signal intensity normalized to the intensity at 25 °C. In (B), lysate samples were treated with increasing DIMP53‐1 concentrations and heated at 39 °C; plot represents the increase in nondenatured p53 calculated setting the signal obtained with DMSO at 39 °C as 0, and the signal obtained with DMSO at 25 °C (considered the maximum amount of nondenatured p53) as 1. Results are mean ± SEM of three independent experiments. (C) DIMP53‐1 concentration–response growth curves in SJSA‐1 and MCF‐7 cells, after 48‐h treatment; data are mean ± SEM of four independent experiments; incubation with DMSO, in equivalent % of DIMP53‐1, was used to normalize the results. (D,E) Cell cycle arrest (D) and apoptosis (E) were determined in SJSA‐1 and MCF‐7 cells at IC_50_ and 2 × IC_50_ (2 × DIMP53‐1) concentrations, after 24‐h treatment; data are mean ± SEM of three independent experiments; values were significantly different from DMSO (*P <* *0.05; **P <* *0.01; ***P <* *0.001). (F) Western blot analysis was performed in SJSA‐1 and MCF‐7 cells, after 24‐h (p21) and 48‐h (PARP, p53, MDM2, BAX) treatments with the IC_50_ of DIMP53‐1 or DMSO. In (A), (B), and (F), immunoblots are representative of three independent experiments; in (B) and (F), GAPDH was used as loading control.

The ability of DIMP53‐1 to block the p53 interaction with MDM2 and MDMX was demonstrated by Co‐IP, in HCT116p53^+/+^ cells. In fact, DIMP53‐1 caused a significant decrease in the amount of MDM2 (at 14 μm) and MDMX (at 7 and 14 μm) co‐immunoprecipitated with p53 (Fig. 2G,H). Based on these results, we attempted to identify the potential molecular target to which DIMP53‐1 would bind, causing the inhibition of the p53 interaction with both MDMs. As DIMP53‐1 is a chemical derivative of the previously reported activator of p53, SLMP53‐1 (Soares *et al*., 2016), we hypothesized that DIMP53‐1 might bind to p53. To confirm this hypothesis, the potential interaction of DIMP53‐1 with p53 was checked by CETSA. In this assay, we analyzed the impact of DIMP53‐1 on p53 thermal stabilization, measured by the amount of soluble p53 upon heating. From 39 °C to 42 °C, 10 μm DIMP53‐1 caused significant p53 thermal stabilization (Fig. 3A). Additionally, DIMP53‐1 induced a concentration‐dependent p53 thermal stabilization, at 39 °C (Fig. 3B), reestablishing the levels of nondenatured p53 protein observed at 25 °C in DMSO‐treated sample. Furthermore, 100 μm DIMP53‐1 (the highest concentration tested to demonstrate the interaction of DIMP53‐1 with p53) did not interfere with MDM2 and MDMX thermal stabilization at different heating temperatures (Fig. S2).

The growth inhibitory effect of DIMP53‐1 was further investigated in human tumor cells expressing wt p53 and overexpressing MDM2 (SJSA‐1 cells) or MDMX (MCF‐7 cells). In these tumor cells, a noticeable growth inhibitory effect was also obtained with DIMP53‐1 (IC_50_ values of 11.8 ± 0.7 μm in SJSA‐1 and 13.3 ± 0.5 μm in MCF‐7) (Fig. 3C). Moreover, it was shown that both in the MDM2‐overexpressing SJSA‐1 cells and in the MDMX‐overexpressing MCF‐7 cells, the growth inhibitory effect of DIMP53‐1 (IC_50_) was mediated by cell cycle arrest (G0/G1‐ and S‐phases in SJSA‐1; G0/G1‐phase in MCF‐7; Fig. 3D) and apoptosis, as evidenced by the increase in Annexin V‐positive cells (Fig. 3E) and PARP cleavage (Fig. 3F). Additionally, in both tumor cell lines, DIMP53‐1 (IC_50_) increased the p53 protein levels and upregulated several p53 transcriptional targets, as demonstrated by the increased MDM2, BAX, and p21 protein levels (Fig. 3F).

Altogether, these results demonstrate that DIMP53‐1 is a selective activator of the p53‐pathway, suppressing the MDM2 and MDMX inhibitory effect in human tumor cells due to a potential interaction with p53.

### 3.3 DIMP53‐1 is nongenotoxic in tumor and normal cells and has low cytotoxicity against normal cells

**Figure 4:**
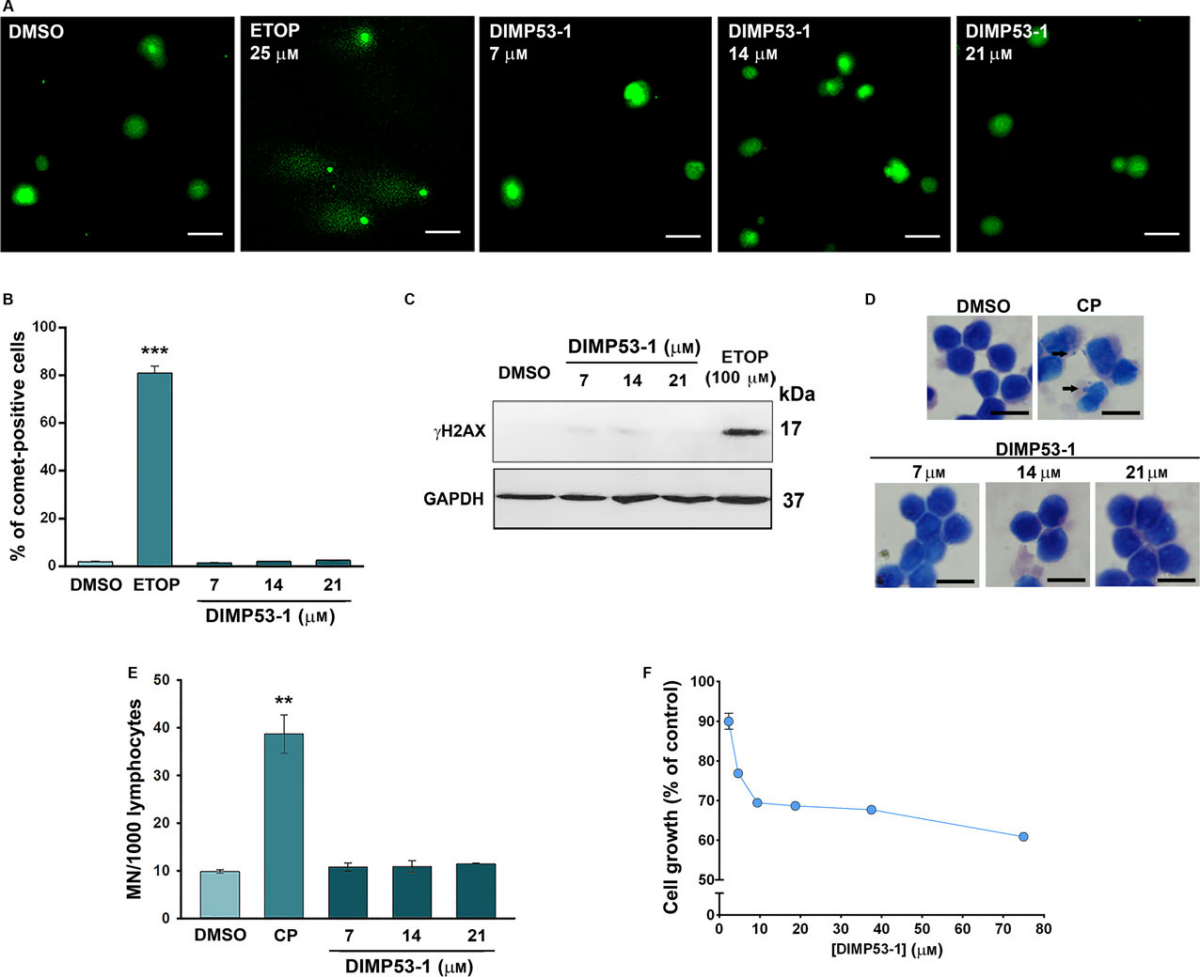
DIMP53‐1 is nongenotoxic in normal and tumor cells and has low cytotoxicity against normal cells. (A–C) DNA damage was measured in HCT116p53^+/+^ cells by comet assay (A and B) and by analysis of γH2AX expression levels (C) after treatment with etoposide (ETOP; positive control) or DIMP53‐1. In (A), scale bar = 20 μm; magnification = 200 ×. In (B), quantification of comet‐positive cells (containing more than 5% of DNA in the tail; assessed by open comet/imagej); 100 cells were analyzed in each group. In (C), γH2AX levels were determined by western blot; immunoblots are representative of three independent experiments; GAPDH was used as loading control. (D and E) Genotoxicity of 7, 14, and 21 μm DIMP53‐1 by cytokinesis‐block micronucleus (MN) assay after 72‐h treatment in human lymphocyte cells; 5 μg·mL^−1^ cyclophosphamide (CP; positive control). In (D), scale bar = 20 μm; magnification = 1000 ×. In (E), the number of MN per 1000 binucleated lymphocytes was recorded. (F) DIMP53‐1 concentration–response growth curve in MCF10A cells, after 48‐h treatment; incubation with DMSO, in equivalent % of DIMP53‐1, was used to normalize the results. In (B), (E), and (F), data are mean ± SEM of three to four independent experiments; in (B) and (E), values were significantly different from DMSO (**P <* *0.01; ***P <* *0.001).

The genotoxicity of DIMP53‐1 was evaluated in tumor and normal cells. In HCT116p53^+/+^ tumor cells, the impact of DIMP53‐1 on DNA damage was analyzed by checking comet‐positive cells and histone H2AX phosphorylated on serine 139 (γH2AX; phosphorylation of histone H2AX marks the first step in cellular response to DNA double‐strand breaks, and its visualization allows the assessment of DNA damage). The results obtained showed that, unlike etoposide (positive control), 7, 14, and 21 μm DIMP53‐1 did not increase the percentage of comet‐positive cells after 48‐h treatment (Fig. 4A,B), or the levels of γH2AX after 12‐h treatment (Fig. 4C). Furthermore, in peripheral lymphocytes of normal individuals, 7, 14, and 21 μm DIMP53‐1 did not increase the number of micronuclei compared to solvent (Fig. 4D,E).

DIMP53‐1 cytotoxicity was also checked in normal cells by assessing its growth inhibitory effect on breast epithelial MCF10A cells, through the SRB assay (Fig. 4F). The IC_50_ value of DIMP53‐1 in these cells was higher than 75 μm, supporting its selective cytotoxicity toward tumor cells.

Altogether, DIMP53‐1 is nongenotoxic in human tumor and normal cells and has low cytotoxic effects against human normal cells.

### 3.4 DIMP53‐1 reduces *in vitro* angiogenesis and tumor cell migration and invasion

**Figure 5:**
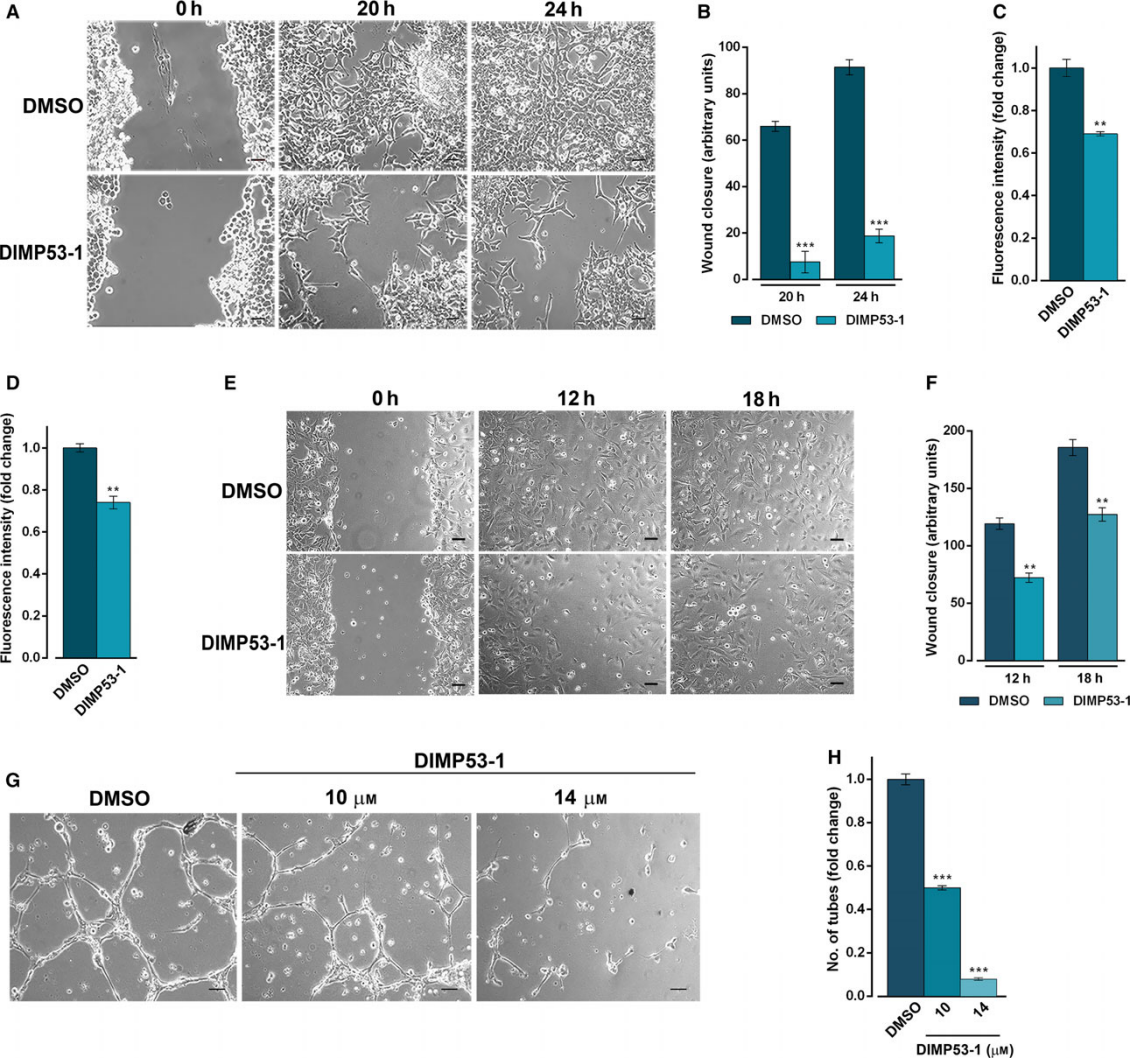
DIMP53‐1 prevents *in vitro* angiogenesis and tumor cell invasion and migration. (A,B) HCT116p53^+/+^ confluent cells treated with 3 μm DIMP53‐1 or DMSO were observed at different time‐points in the wound healing assay. (B) Quantification of wound closure of HCT116p53^+/+^ cells in five randomly selected microscopic fields. (C) Effect of 3 μm of DIMP53‐1 on the migration of HCT116p53^+/+^ cells for 24 h, analyzed by the chemotaxis assay. (D) Effect of 3 μm of DIMP53‐1 on the invasion of HCT116p53^+/+^ cells for 48 h, analyzed by cell invasion assay. In (C) and (D), migratory cells were quantified by fluorescence signal; fold changes are relative to DMSO and correspond to mean ± SEM of three independent experiments. (E,F) HMVEC‐D endothelial confluent cells treated with 14 μm of DIMP53‐1 or DMSO were observed at different time‐points in the wound healing assay. In (F), quantification of wound closure of HMVEC‐D endothelial in five randomly selected microscopic fields. (G) Antiangiogenic effect of 10 and 14 μm of DIMP53‐1 was evaluated in HMVEC‐D cells for 16 h by the endothelial cell tube formation assay. (H) Quantification of tube‐like structures in five randomly selected microscopic fields; fold changes are relative to DMSO and correspond to mean ± SEM of three independent experiments. In (A), (E), and (G), scale bar = 5 μm and magnification = 100 ×. In (B), (C), (D), (F), and (H), values were significantly different from DMSO (**P <* *0.01; ***P <* *0.001).

The impact of DIMP53‐1 on HCT116p53^+/+^ cell migration and invasion was investigated. With such purpose, the concentration of 3 μm (IC_10_) of DIMP53‐1, which does not significantly interfere with HCT116p53^+/+^ cell growth, was used. In the wound healing assay, 3 μm of DIMP53‐1 significantly inhibited HCT116p53^+/+^ cell migration, and the subsequent wound closure, when compared to solvent (Fig. 5A,B). These results were confirmed using the chemotaxis cell migration assay, in which 24‐h treatment with 3 μm of DIMP53‐1 led to a significant reduction in HCT116p53^+/+^ cell migration compared to solvent (Fig. 5C). Additionally, 3 μm of DIMP53‐1 inhibited HCT116p53^+/+^ cell invasion through a Matrigel^®^ matrix, evaluated by the cell invasion assay after 48‐h treatment (Fig. 5D).

The antiangiogenic potential of DIMP53‐1 was also investigated. Initially, its antiproliferative effect on HMVEC‐D endothelial cells was assessed, and an IC_50_ higher than 50 μm was obtained (data not shown), indicating low toxicity of DIMP53‐1 toward endothelial cells. Thereafter, the wound healing assay was performed to evaluate the effect of 14 μm of DIMP53‐1 (IC_10_ value in HMVEC‐D) on HMVEC‐D cell migration. At this concentration, a significant decrease in endothelial cell migration was observed (Fig. 5E,F). Moreover, using an *in vitro* angiogenesis assay, a significant antiangiogenic effect was observed after 16‐h treatment with 10 and 14 μm of DIMP53‐1. In fact, DIMP53‐1 led to a dose‐dependent decrease in HMVEC‐D tube formation (Fig. 5G,H).

Altogether, DIMP53‐1 demonstrates *in vitro* antiangiogenic, antimigration, and anti‐invasive effects.

### 3.5 DIMP53‐1 has *in vivo* antitumor activity without apparent toxic side effects

To evaluate some primary toxicity signs, 50 mg·kg^−1^ DIMP53‐1 was tested in Wistar rats. Following the same administration procedure conducted in tumor xenograft mice models, organs’ relative weight (trophism) and biochemical and hematological data were analyzed for saline, vehicle, and DIMP53‐1 groups (Table 1). No differences between the three groups on relative weight of liver, kidneys, heart, and spleen were observed. Concerning biochemical data, only a slight decrease in urea in the vehicle group compared to the saline group, and a slight increase in uric acid in the DIMP53‐1 group compared to controls (saline and vehicle groups) were observed. These results indicated no apparent liver or kidney toxicity. Regarding hematological data, just a small increase in reticulocyte number was observed in the vehicle group compared to the saline group, with no alterations between DIMP53‐1 and the control groups. Overall, no apparent toxic side effects were observed for DIMP53‐1 on the tissues most commonly affected by conventional chemotherapeutics.

**Table 1:** Toxicity studies of DIMP53‐1 in Wistar rats

	Saline	Vehicle	Treated
Body weight and relative tissue weight (trophism)			
BW (g)	340.30 ± 13.45	361.80 ± 0.01	353.25 ± 3.99
Heart/BW (g·kg^−1^)	3.03 ± 0.03	3.01 ± 0.09	3.19 ± 0.12
Liver/BW (g·kg^−1^)	38.71 ± 2,36	38.52 ± 0.89	40.72 ± 1.14
Kidney/BW (g·kg^−1^)	7.08 ± 0.40	6.81 ± 0.27	6.96 ± 0.32
Spleen (g·kg^−1^)	2.10 ± 0.20	2.15 ± 0.12	2.07 ± 0.20
Biochemical data			
Blood glucose (mg·dL^−1^)	188.0 ± 7.51	207.20 ± 7.00	242.25 ± 25.68
Urea (mg·dL^−1^)	20.67 ± 0.77	18.34 ± 0.24*	19.10 ± 0.62
Uric acid (mg·dL^−1^)	1.07 ± 0.07	0.82 ± 0.10	4.10 ± 0.50*
Creatinine (mg·dL^−1^)	0.30 ± 0.00	0.30 ± 0.01	0.36 ± 0.03
Total proteins (g·dL^−1^)	5.50 ± 0.00	5.35 ± 0.17	6.38 ± 0.40
Albumin (g·dL^−1^)	3.03 ± 0.03	2.90 ± 0.05	3.13 ± 0.03
ALT (U·L^−1^)	36.00 ± 2.89	30.75 ± 3.30	35.00 ± 1.87
AST (U·L^−1^)	95.33 ± 25.36	56.25 ± 4.60	115.75 ± 20.89
Total cholesterol (mg·dL^−1^)	44.33 ± 1.76	52.40 ± 3.54	54.00 ± 3.37
Triacylglycerols (mg·dL^−1^)	107.00 ± 16.29	161.40 ± 17.55	229.50 ± 26.70
Hematological data			
RBC count (×10^6 ^μL^−1^)	7.10 ± 0.15	7.41 ± 0.28	8.23 ± 0.24
HGB (g·dL^−1^)	13.97 ± 0.18	13.95 ± 0.46	14.68 ± 0.28
HCT (%)	40.50 ± 0.82	42.28 ± 2.03	47.85 ± 1.87
WBC counts (×10^3 ^μL^−1^)	1.93 ± 0.68	2.15 ± 0.88	1.05 ± 0.26
PLT counts (×10^6 ^μL^−1^)	811.00 ± 29.61	793.80 ± 28.35	726.75 ± 47.43
RET counts (%)	2.80 ± 0.12	3.56 ± 0.23*	3.59 ± 0.31

Data were analyzed for saline, vehicle, and 50 mg·kg^−1^ DIMP53‐1 (treated) rat groups, after four intraperitoneal administrations (twice a week). Results are mean ± SEM of four independent experiments; *P < 0.05 (comparison was made between saline and vehicle groups, and between vehicle and treated groups). ALT, alanine aminotransferase; AST, aspartate aminotransferase; BW, body weight; CK, creatine kinase; HCT, hematocrit; HGB, hemoglobin concentration; PCT, plateletcrit; PLT, platelet; RBC, red blood cell count; RET, reticulocytes; WBC, white blood cells.

**Figure 6:**
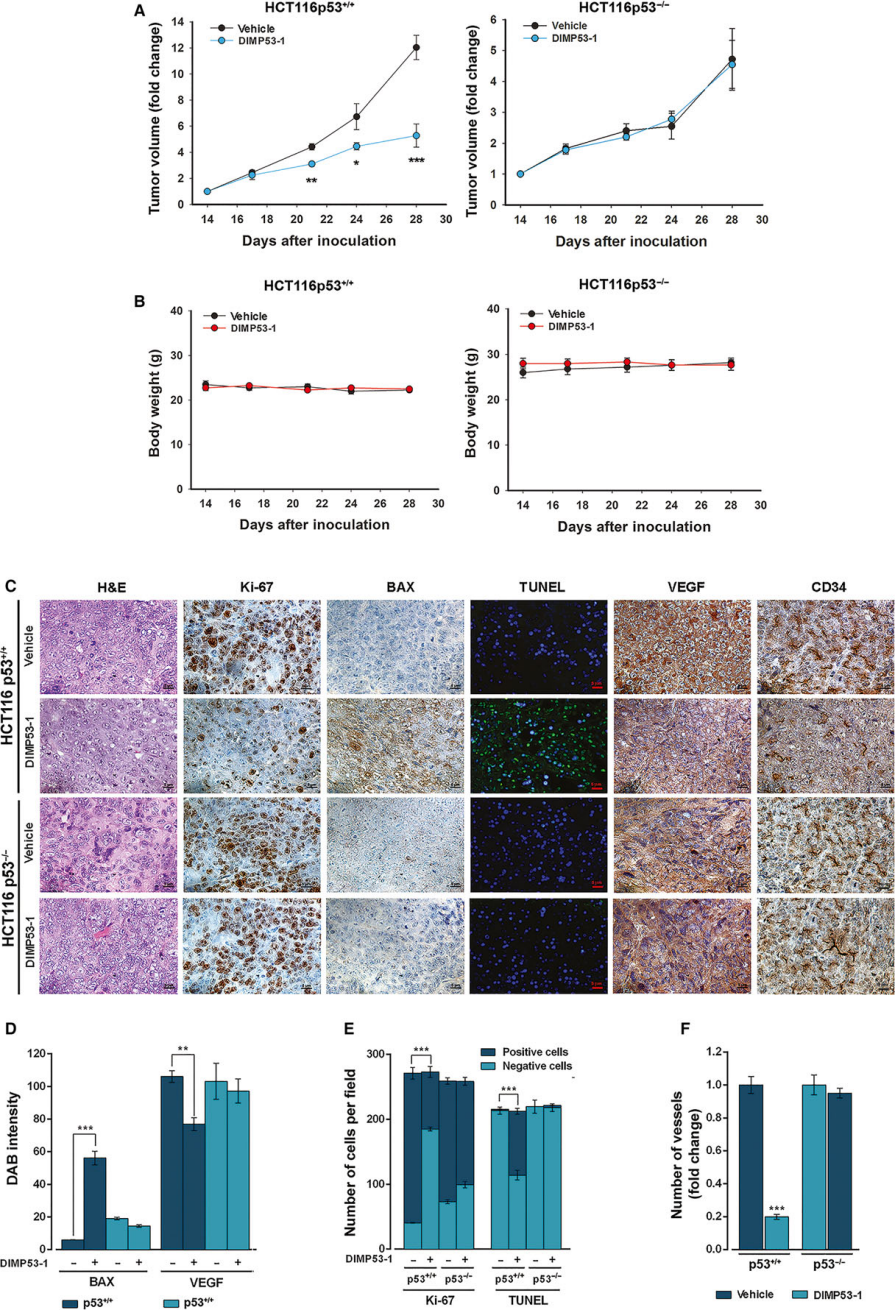
DIMP53‐1 has *in vivo* p53‐dependent antitumor activity by inducing apoptosis and inhibiting proliferation and angiogenesis. BALB/c nude mice of about 10 weeks were inoculated subcutaneously, in the dorsal flank, with HCT116p53^+/+^ and HCT116p53^−/−^ tumor cells; when tumors reached approximately 100 mm^3^ volume (14 days after the grafts), mice were treated twice a week with 50 mg·kg^−1^ DIMP53‐1 or vehicle (control) by intraperitoneal injection for two weeks. (A) Tumor volume growth curves of mice carrying p53^+/+^ or p53^−/−^ HCT116 xenografts treated with DIMP53‐1 or vehicle; data are mean ± SEM of the tumor volume fold change to the start of treatment. (B) Mice body weight during treatment with DIMP53‐1 or vehicle; values were not significantly different from vehicle (*P *>* *0.05). (C) Representative images of Ki‐67, BAX, DNA fragmentation (TUNEL), CD34, and VEGF detection in p53^+/+^ and p53^−/−^ HCT116 xenograft tumor tissues treated with DIMP53‐1 or vehicle at the end of treatment (scale bar = 5 μm; magnification = 400×); H&E (hematoxylin and eosin). (D–F) Quantification of immunohistochemistry of p53^+/+^ and p53^−/−^ HCT116 xenograft tumor tissues treated with DIMP53‐1 or vehicle. In (D), BAX and VEGF staining quantification by evaluation of DAB (3,3′‐diaminobenzidine) intensity. In (E), quantification of the number of positive and negative Ki‐67 and TUNEL cells. In (F), evaluation of microvessel density by quantification of vessels stained with anti‐CD34; data are mean ± SEM of the number of vessels per mm^2^ fold change to the vehicle. In (A), (D), (E), and (F), values were significantly different from vehicle (*P <* *0.05; **P <* *0.01; ***P <* *0.001).

The *in vivo* antitumor potential of DIMP53‐1 was evaluated using human tumor xenograft mice models of p53^+/+^ and p53^−/−^ HCT116 cells. Four intraperitoneal administrations of 50 mg·kg^−1^ DIMP53‐1 inhibited the growth of p53‐expressing HCT116 tumor compared to vehicle (Fig. 6A). Conversely, for the same conditions, DIMP53‐1 did not interfere with the growth of p53‐null HCT116 tumors, further reinforcing its p53‐dependent antitumor activity (Fig. 6A). Furthermore, no significant body weight loss or morbidity signs were observed in DIMP53‐1‐treated mice compared to vehicle (Fig. 6B).

The subsequent analysis of tumor tissues was performed to check *in vivo* p53‐dependent antitumor events promoted by DIMP53‐1. Proliferation, apoptosis, and angiogenesis markers were checked in p53^+/+^ and p53^−/−^ HCT116 tumor tissues by immunohistochemistry and TUNEL staining (Fig. 6C–F). In p53‐expressing tumor tissues, DIMP53‐1 reduced proliferation (decrease in Ki‐67‐positive staining) and stimulated apoptosis (increase in BAX expression and DNA fragmentation demonstrated by TUNEL‐positive staining), compared to vehicle (Fig. 6C–E). To study the angiogenic profile of tumor tissues, the vascular endothelial growth factor (VEGF; angiogenesis‐inducing factor) and the microvessel density (MVD; determined using the marker of newly formed vessels CD34) were determined. The results obtained revealed lower levels of VEGF and MVD in p53‐expressing tumor tissues treated with DIMP53‐1, compared to vehicle (Fig. 6C,D,F). Particularly, an overall fivefold reduction in MVD was observed in p53‐expressing tumor tissue treated with DIMP53‐1 (Fig. 6F). Conversely, no apparent differences in these markers were observed between DIMP53‐1 and vehicle in p53‐null tumors (Fig. 6C–F).

Altogether, these results support an *in vivo* p53‐dependent antiproliferative, proapoptotic, and antiangiogenic activity of DIMP53‐1.

## 4 Discussion

The complexity underlying cancer highlights the need for the development of strategies that would target the intricate cancer network, in order to modulate the different hallmarks of cancer and hence treat cancer as a complex disease. The key role of p53 in cancer hallmarks renders p53‐targeted anticancer therapies highly encouraging. Actually, an effective inhibition of cancer development and progression has been achieved with strategies devised to rectify a dysfunctional p53 pathway, particularly due to MDM2 and MDMX overexpression (Hong *et al*., 2014; Li and Lozano, 2013; Wade *et al*., 2013). Consistently, the dual inhibition of the p53–MDM2/X interactions, for full p53 reactivation, has gained strength to treat wt p53‐expressing tumors, particularly MDMX‐overexpressing tumors commonly resistant to only MDM2 inhibitors (Burgess *et al*., 2016; Li and Lozano, 2013).

Here, we report a novel tryptophanol‐derived oxazoloisoindolinone, DIMP53‐1, identified as a new dual inhibitor of the p53–MDM2/X interactions. The molecular mechanism of action of DIMP53‐1, identified in yeast, was validated in human tumor cells with and without p53. In fact, DIMP53‐1 caused tumor cell growth inhibition mediated by p53 stabilization and upregulation of p53 transcriptional targets involved in cell cycle arrest and apoptosis, in wt p53‐expressing tumor cells, including MDM2‐ or MDMX‐overexpressing cells. Notably, DIMP53‐1 inhibited the p53–MDM2/X interactions by potentially binding to p53 in human tumor cells.

The loss of p53 function has been related to the development of a metastatic phenotype (Powell *et al*., 2014), the most frequent cause of mortality in patients with cancer (Cordani *et al*., 2016; Spano *et al*., 2012). Actually, p53 stimulates the transcription of repressors of cell migration and invasion (Powell *et al*., 2014). It is therefore expected that the restoration of p53 function may suppress cancer dissemination. Thus, an effective antimigratory and anti‐invasive activity of DIMP53‐1 was demonstrated in wt p53‐expressing tumor cells.

Angiogenesis is a major hallmark of cancer as proliferation and metastatic spread of cancer cells depend on the adequate supply of oxygen and nutrients (Baeriswyl and Christofori, 2009). Actually, several antiangiogenic agents have been explored in cancer treatment, particularly in combination with conventional chemotherapeutic agents (Vasudev and Reynolds, 2014). The p53 activity has been negatively correlated with this process through indirect inhibition of key proteins, such as VEGF. Particularly, it was demonstrated that p53 indirectly represses VEGF expression by inhibiting transcription factors such as SP1 and E2F (Pal *et al*., 2001; Qin *et al*., 2006). Here, the therapeutic potential of DIMP53‐1 was further reinforced through confirmation of *in vivo* antiangiogenic activity through depletion of VEGF in tumors. Interestingly, despite the antiangiogenic activity of DIMP53‐1 in an *in vitro* endothelial cell system (without tumor cells), the results obtained *in vivo* showed that this antiangiogenic effect is highly dependent on tumor environment, particularly of the p53 status in these tumors. In fact, the antiangiogenic activity of DIMP53‐1 was suppressed in p53‐null tumor xenografts. These results emphasize a strong connection between the activation of the p53 pathway by DIMP53‐1 and its antiangiogenic activity in tumors. Further studies are required to clarify the molecular pathways involved in DIMP53‐1 antiangiogenic activity.

Additionally, DIMP53‐1 is nongenotoxic in both normal and tumor cells and presents no significant toxicity both in normal cells and in rats. Most importantly, in human tumor xenograft mice models, a p53‐dependent antitumor activity of DIMP53‐1 was observed. Actually, DIMP53‐1 suppressed the growth of wt p53‐expressing tumors, through inhibition of proliferation and induction of apoptosis, without interfering with the growth of p53‐null tumor xenografts.

Interestingly, to date, only the furan derivative RITA was reported as a small‐molecule inhibitor of the p53–MDM2 interaction by binding to p53 instead of MDM2 (Gomes *et al*., 2016; Hong *et al*., 2014; Wade *et al*., 2013). However, conversely to RITA, DIMP53‐1 has no genotoxic effects, also acts on the p53–MDMX interaction, and exhibits selectivity to the p53 pathway, particularly highlighted in *in vivo* mice models.

## 5 Conclusions

This work reports the identification of a new potential p53 ligand, which activates the p53 function through inhibition of its interaction with MDM2/X. DIMP53‐1 has *in vivo* p53‐dependent antitumor properties with no apparent toxic side effects, and exhibits antiproliferative, proapoptotic, antiangiogenic, anti‐invasive, and antimigratory activities. Collectively, although DIMP53‐1 is a targeted agent, it also presents a multifunctional activity, interfering with several hallmarks of cancer. Besides its great promise as an anticancer drug candidate, DIMP53‐1 is also an encouraging starting point for further development of dual inhibitors of the p53–MDM2/X interactions with improved therapeutic potential for clinical translation.

## Author contributions

JS performed experiments, analyzed and interpreted the data, and wrote the manuscript; ME performed the synthesis and characterization of DIMP53‐1; LR, HR, ASG, SG, JBL, and AI performed experiments, analyzed and interpreted the data; FR and CG performed the *in vivo* experiments; MMMS conceived the design and synthesis of DIMP53‐1, analyzed and interpreted the data; LS conceived the study, analyzed and interpreted the data, and wrote the manuscript. All authors read and approved the final version of the manuscript.

## Supporting information


**Scheme S1.** Synthesis of DIMP53‐1.
**Fig. S1.** ^1^H NMR and ^13^C NMR data for compound DIMP53‐1.
**Fig. S2.** CETSA experiments of MDM2 and MDMX.
