# EXPRESSION OF CONCERN: DIMP53‐1: A Novel Small‐Molecule Dual Inhibitor of p53–MDM2/X Interactions with Multifunctional p53‐Dependent Anticancer Properties

**DOI:** 10.1002/1878-0261.70284

**Published:** 2026-06-03

**EXPRESSION OF CONCERN**: J. Soares, M. Espadinha, L. Raimundo, H. Ramos, A. S. Gomes, S. Gomes, J. B. Loureiro, A. Inga, F. Reis, C. Gomes, M. M. M. Santos, and L. Saraiva, “DIMP53‐1: A Novel Small‐Molecule Dual Inhibitor of p53–MDM2/X Interactions with Multifunctional p53‐Dependent Anticancer Properties,” *Molecular Oncology* 11, no. 6 (2017): 612–627, https://doi.org/10.1002/1878-0261.12051.

This Expression of Concern is for the above article, published online 10 March 2017 in Wiley Online Library (wileyonlinelibrary.com), and has been issued by agreement between the journal Editor‐in‐Chief, Kevin M. Ryan; the Federation of European Biochemical Societies; and John Wiley & Sons Ltd. The Expression of Concern has been agreed due to concerns raised by third parties. Specifically, image duplication has been identified within Figure 6C. Further investigation by the journal has also identified that the same loading control blot has been presented across Figures 2E and 3E in different scientific contexts. The authors recognize the importance of providing comprehensive support for these figures and are in the process of providing additional data to further substantiate the reported results. Therefore, the journal has decided to issue an Expression of Concern to inform and alert the readers while the authors undertake the experimental work needed to address the identified issues.

